# Instructor facilitation mediates students’ negative perceptions of active learning instruction

**DOI:** 10.1371/journal.pone.0261706

**Published:** 2021-12-23

**Authors:** Elizabeth S. Park, Ashley Harlow, Amir AghaKouchak, Brigette Baldi, Nancy Burley, Natascha Buswell, Roderic Crooks, Darren Denenberg, Peter Ditto, Kimberley Edwards, Mariana Garcia Junqueira, Andrew Geragotelis, Amanda Holton, Joel Lanning, Rachel Lehman, Audrey Chen, Alessandra Pantano, Jenny Rinehart, Mark Walter, Adrienne Williams, Jennifer Wong-Ma, Michael Yassa, Brian Sato

**Affiliations:** 1 Education Research Initiative, University of California Irvine, Irvine, California, United States of America; 2 Center for Teaching and Learning, University of Georgia, Athens, Georgia, United States of America; 3 School of Biological Sciences, Education Research Initiative, University of California Irvine, Irvine, California, United States of America; University of Geneva, SWITZERLAND

## Abstract

Studies have demonstrated students’ resistance to active learning, despite evidence illustrating that their learning is improved relative to students in lectures. Specifically, while active learning and group work are effective at engaging students in their learning process, studies report that students’ perceptions of active learning approaches are not always positive. What remains underexplored is whether students’ perceptions of active learning improve with effective instructor facilitation and whether there exists differential perceptions between racially minoritized students and represented students. Here, we estimate students’ perceptions of effective instructor facilitation as the mediator in the relationship between active learning and perceptions of learning and perceived utility for class activities (task value). Then, we examine differences by racial identification. We collected classroom observation data to empirically categorize courses as active learning or lecture-based and surveyed 4,257 college students across 25 STEM classrooms at a research-intensive university. We first examined the relationship between active learning on student perceptions and found a negative relationship between active learning and perceptions of learning and task value for both racially minoritized students and represented students. Next, we assessed whether students’ perceptions of instructor effectiveness in facilitating group activities mediate these negative relationships. We found that, on average, students of all races were more likely to positively perceive instructor facilitation in active learning classes relative to lectures. In turn, the positive perceptions of instructor facilitation partially suppressed the negative relationship between active learning and perceptions of learning and task value. These results demonstrate that effective instructor facilitation can influence both students’ self-assessment of learning and perceived utility of the learning activities, and underscores the importance of developing pedagogical competence among college instructors.

## Introduction

Active learning instruction is characterized by increased student engagement, frequent assessment of conceptual learning, and group activities [1–3]. In particular, these group activities are often a defining feature of active learning instruction [4] and are linked to deeper learning in higher education [5]. Studies have found that active learning instruction better engages college students in Science, Technology, Engineering, and Mathematics (STEM) classrooms [6] and improves learning outcomes [7–9]. Moreover, active learning instruction may decrease academic performance differences between racially minoritized students—defined as African-American, Latino/Latina, Native American, Southeast Asians or Pacific Islander/Native Hawaiian—and represented students [10]. Despite these collective benefits, active learning instruction has not been widely adopted in college settings [11, 12]. A contributing factor as to why faculty are hesitant to implement active learning is the perception that their students are resistant to active learning instruction [13, 14] with variable findings regarding student receptiveness to active learning pedagogies [6, 15, 16]. Furthermore, recent evidence found that students taught using active learning approaches reported lower perceptions of learning than their peers in lecture-based classrooms despite exhibiting greater learning in the course [17]. Relatedly, while it has been shown that racial gaps in academic performance decrease in active learning courses [10], we know less about whether racially-minoritized students’ perceptions of learning vary from that of their more represented peers. While all students in active learning classrooms face increased academic accountability, racially minoritized college students may face additional stress in an environment where their racial or college-going identit(ies) are less represented. If racially minoritized students are cued to feel out of place or feel uncomfortable as fewer students of similar racial backgrounds are present in class, they face an undue burden to fit in [18–20]. In contrast, racial performance gaps in active learning instruction relative to lecture-based instruction may decrease as racially minoritized students are given more feedback on their learning and are provided with scaffolded time on tasks [10].

Perceptions of learning are directly connected to student success over time, as students who hold accurate perceptions of learning in the course while also believing that the course material is useful and important are more likely to exert effort and persist in STEM fields [21, 22]. Prior research has documented that misperceptions about learning led STEM students to switch to non-STEM majors relative to students who held more accurate perceptions about the major [21]. Similarly, college students’ motivation to engage with the material and exert cognitive effort hinge on their perceptions of whether the activities are perceived as useful and important to their learning [22, 23]. Yet, inaccurate perceptions become most pronounced when the context and tasks are unfamiliar or unstructured [24], which can include active learning practices as these pedagogies are exceptions rather than the norm [12]. In active learning classrooms, therefore, instructor facilitation can shape students’ perceptions of learning and whether they believe the in-class activity is useful to their educational progress.

Indeed, students’ perceptions of learning may not be positive when instructors do not effectively facilitate group activities [14]. Prior research has shown that without proper instructor facilitation, students do not equally contribute to group assignments and tend to not assume group roles in intended ways [25, 26]. In turn, students may hold inaccurate perceptions of their learning progress and perceive that their learning tasks have lower value as they regard active learning as disjointed and lacking in flow relative to well-organized lecture-based instruction [17].

Our study is motivated by three related questions: (1) do students in active learning classrooms differ on perceptions of learning/task value than students in lecture-based classrooms, (2) do these perceptions differ for racially minoritized students, and (3) are these relationships mediated by students’ perceptions of effective instructor facilitation? We contribute to the literature along the following three dimensions. By answering these questions, we place focus on instructors’ behaviors and students’ perceptions of instructor behavior in active learning classrooms. Through a focus on instructional practices, we are able identify potential misalignment between faculty and students’ perceptions in active learning classrooms. Second, we analyze whether perceptions differ among racially-minoritized students relative to represented students. A closer look at the promise of a certain instruction type is both policy-relevant and timely given the need to better engage and support racially-minoritized students. Third, previous related studies that focused on perceptions of learning have limited generalizability given that they examined sections of a particular course in one discipline [8, 17, 27]. Our study leverages survey data of students in multiple courses across various STEM disciplines at a large public institution and offers a broader examination of students’ perceptions of learning and task value, enabling us to better understand and generalize these patterns.

## Materials and methods

### Data collection

We conduct our examination using data collected across 25 introductory STEM classrooms offered during fall 2019 or winter 2020 at a large public, research-intensive institution. The Institutional Review Board Office (IRB) at the institution where this study was conducted has a self-exempt policy for Exempt Category 1 and 2 research, which this work falls under, and thus the study was not formally reviewed by the IRB. Faculty and students were informed of the work with a study information sheet that specified an opt-out policy. Any individuals not willing to participate in the research contacted a third-party through email and their data was removed for analysis purposes. All data obtained for this study were de-identified. We are able to characterize classes as active learning or lecture-based using rich classroom observation data obtained using the Classroom Observation Protocol for Undergraduate STEM (COPUS) protocol [12, 28]. COPUS is a classroom observation protocol in which observers record instructor and student behavior during every two minutes of a class period [28]. The Teaching and Learning Center at this institution selected the classes based on the following criteria: undergraduate lecture classes (excluding lab sections, discussions, and seminar courses) held in rooms with capacity for 60 students or greater. We grouped classes as instruction with high group activity (i.e, active learning) and instruction with low group activity (i.e., lecture-based) using the COPUS data and k-means cluster analysis. The goal of the k-means cluster analysis is to decrease the number of within sums of squared errors of a cluster by minimizing the distance from the centroid while maximizing the distance between clusters [29, 30].

**Fig 1:**
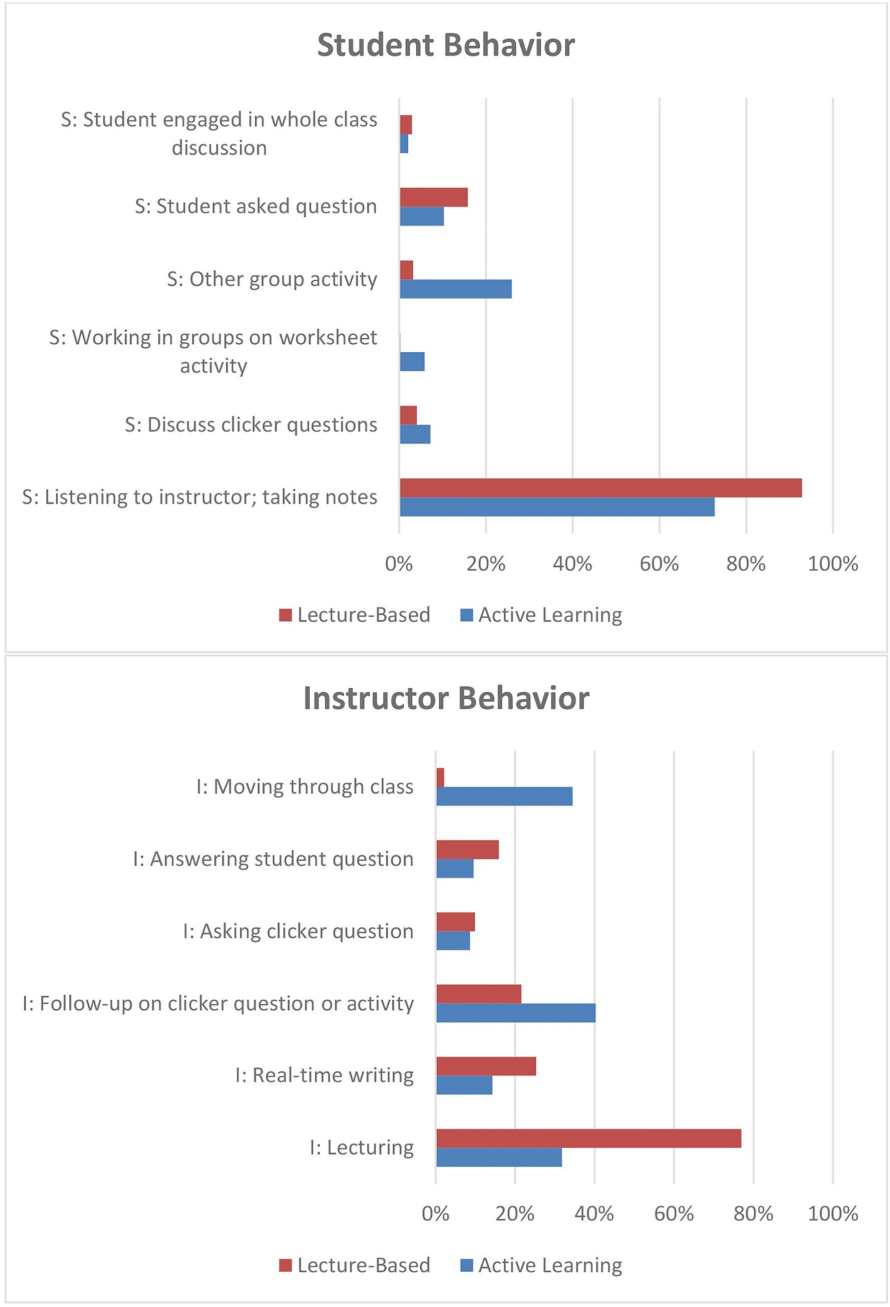
Distribution of select student and instructor COPUS codes in active learning and lecture-based classrooms.

Fig 1 displays the distribution of recorded instructor and student behaviors in the 25 classes, categorized as either active learning or as lecture-based. In active learning classes, instructors spent less time on lecturing and more time moving through class compared to low group activity courses. For example, in active learning classes, instructors spent, on average, 32% of the two-minute intervals lecturing and 34% moving through class and students spent 26% working in groups (blue bar, n = 15). In lecture-based class, instructors spent, on average, 77% of the two-minute intervals lecturing and 2% moving through class and students spent 3% of the two-minute intervals working in groups (red bar, n = 10).

All instructors who participated in the classroom observations were also invited to distribute a student survey and participate in a faculty survey during the last two weeks of the term. In the survey, we asked students about their perceptions of instructor effectiveness in facilitating group activities, task value, and their perceptions of learning (see S1 Appendix for survey items). Student survey response rate across the 25 classes ranged from 67% to 100% for a total sample size of 4,257 student respondents. After we completed survey data collection, we obtained administrative data that include students’ demographic and achievement records. We merged in the administrative data with survey data to create a detailed dataset with student survey responses and their background characteristics.

### Empirical analysis

We estimate a series of regression models to examine whether students taught with high or low group activities differ on perceptions of learning and task value and to estimate the mediating effect. We calculate the extent to which the mediator explains the relationship between instruction type and outcomes by comparing the direct effect to the total effect [31]. If the total effect is reduced to zero once the mediator is included in the model, we conclude that full mediation has occurred. If the magnitude of the coefficient is reduced once we account for the mediator, we conclude that partial mediation has occurred. In contrast, if the magnitude of the coefficient becomes larger once we account for the mediator, we conclude that the mediator has suppressed the relationship [32].

We first estimate the relationship between instruction type on the mediator, perceptions of instructor effectiveness in facilitating group activities. Next, we estimate the direct effect of the instruction type on student outcomes controlling for the mediator. To examine whether any patterns we observe vary by race, we estimate a moderated mediation analysis [33]. The moderated mediation analysis was conducted to examine whether the strength and the direction of the mediation effect differ for racially-minoritized students. Specifically, we interact the previous models by race to tease out differential effects on the mediator and outcomes. To obtain our moderated mediation estimates, we estimate the moderation of the treatment effect on the mediator and multiplied that with the moderation of mediating effect on the outcome accounting for the treatment effect. We use R version 4.0.3 mediation package to conduct all of our mediation analyses [34]. We estimate the standard errors and confidence interval of the mediation effect using bootstrap standard errors resampled 1000 times [34, 35].

We include a number of carefully chosen covariates to account for the fact that we are comparing STEM courses that may differ across a number of dimensions. To eliminate confounding variables such as student-level differences or instructor-level differences, we include student demographic characteristics such as students’ major (STEM versus non-STEM), gender, race, low-income status, first-generation status, high school GPA, SAT math, SAT verbal, and whether students transferred from another university. Furthermore, we control for classroom-level characteristics because students’ perceptions of group activities may be influenced by the peer composition of the class as well as by the instructor [36]. We created various measures like the average high school achievement of students in the class and the proportion of racially-minoritized students in the class. In addition, we hold constant the class enrollment number because class size is a predictor of student engagement [37] as well as whether the class was offered in a building that is designed to encourage group activities and discussion on campus as the infrastructure may influence student perceptions [38]. We also administered a short survey to instructors and obtained information on their prior teaching experiences and teaching self-efficacy. Thus, we were able to control for prior teaching experiences, instructor teaching self-efficacy, faculty gender, and faculty rank (i.e., Lecturer, Assistant, Associate, etc.). Finally, we account for grading differences in each class to account for the possibility that one instructor may grade harder than another instructor teaching the same class. We create this indicator by pulling student-level administrative data of all the classes in our analytic sample from 2016 and beyond. Then, we averaged students’ performance of prior terms at the instructor-by-class level (i.e., determine classroom-specific average grade taught by a particular instructor). For instructors who taught the course for the first time within the timeframe of our data, we included the average performance of the class they currently taught.

In addition to a rich set of covariates, we include several fixed effects to account for any common group-level differences. For instance, we include a time trend to account for when the students took the survey and for differences from term-to-term (e.g., common shock occurring at a particular term). We also include entry term fixed effects to compare students from the same entering cohort. Lastly, we control for departmental differences by including department fixed effects, thereby comparing courses within the same department. We cluster the standard errors at the classroom-by-term level as there may be autocorrelation in student survey responses due to being in the same class during a particular term. Therefore, we hold constant a large number of potentially confounding factors such as departmental differences and term-by-term fluctuations, differences in the types of students in the classroom (i.e., demographic differences and prior achievement), and instructor differences (i.e., prior teaching experience, instructor rank etc.). Despite the inclusion of these covariates, we note that our analysis is necessarily descriptive and the aim of our analysis is to provide a descriptive look at active learning instruction, capitalizing on the availability of rich data.

## Results

### Demographics

A majority (75%) of the students in our analytic sample are STEM majors, half of them are first-generation college students and a fifth of them are transfer students. Compared to the broader student population enrolled at this large, selective institution during fall 2019, a greater proportion of students in our analytical sample were STEM majors (76% in our sample versus 47%). In addition, a greater proportion of students in our analytical sample are identified as racially-minoritized students relative to all students enrolled at this institution (54% in our sample versus 29%). Despite these notable differences, we note similarities with regards to the representation of transfer students as well as the proportion of low-income or first-generation students (Table 1).

**Table 1:** Generalizability table.

	Fall 2019 Undergraduate Enrollment	Analytic Sample	
	M	M	SD
STEM Major	47%	76%	
Women	52%	57%	
Racially Minoritized	29%	54%	
Transfer Student	22%	20%	
Low-Income	38%	34%	
First Generation	47%	50%	
High School GPA		3.91	0.45
SAT Math		626.26	96.87
SAT Verbal		583.26	92.42

There were 30,382 first-time undergraduate students enrolled at this institution and 4,257 students in the analytical sample.

We next present various student-, course-, or instructor-level characteristics. Students in active learning classes are more likely to be women and major in STEM, and have entered college with higher high school GPA than students in low group activity courses (Table 2). Moreover, we see instructor-level differences between those who used active learning instructional approaches relative to instructors who used mostly lectures, in that fewer first-time instructors tend to use active learning approaches. Specifically, close to 20% of instructors who used mostly lectures also reported having no prior experience teaching the course. In contrast, only 6% of instructors who taught using active learning approaches reported that this is their first time teaching the course. Regardless of prior experience, however, instructors in both instruction types rated themselves highly on their teaching self-efficacy (5.64 on a 7-point scale). As previously mentioned, these covariates are included as control variables in all of the analyses.

**Table 2:** Student characteristics and class composition.

	Active Learning	Lecture-Based	
Variable	M or %	M or %	p-value
*Student Characteristics*			
STEM Major	88%	62%	0.000
Women	59%	56%	0.008
Racially Minoritized	54%	55%	0.751
Transfer Student	17%	24%	0.000
Low-income	33%	35%	0.704
First Generation	47%	53%	0.000
Weighted High School GPA	3.94	3.88	0.000
SAT Math	630.38	620.81	0.021
SAT Verbal	584.68	581.39	0.503
*Class-Level Measures*			
% URM	49%	51%	0.860
% Women	51%	53%	0.885
% Low-Income	30%	33%	0.831
Average SAT Math	563.49	532.39	0.526
Average SAT Verbal	513.71	491.73	0.634
Average High School GPA	3.92	3.85	0.226
Active Learning Building	80%	40%	0.105
*Instructor Characteristics*			
Women	67%	60%	0.799
Lecturer	7%	30%	0.285
Assistant	40%	40%	0.962
Associate	13%	10%	0.663
Full	40%	20%	0.444
No prior experience	7%	20%	0.503
Prior experience teaching course	47%	60%	0.565
Prior experience teaching course in active learning infrastructure	47%	20%	0.234
Instructor self-efficacy	5.55	5.57	0.717

There are 2,366 students in active learning courses and 1,902 students in lecture-based courses.

### Student perceptions of learning and task value

To address our first research question, we estimate the relationship between instruction type and student perceptions using student survey data. Students’ perceptions of learning were assessed with the survey question, *I feel like I learned a great deal in the course* [17]. Table 3 panel A indicates that students in active learning classrooms, on average, responded 9.5 percentage points lower (*p <* 0.001) on perceptions of learning than students in lecture-based classrooms. 77% of students in low group activity classrooms agreed or strongly agreed that they learned a great deal whereas 67.5% of students in high group activity classrooms felt like they learned a great deal in the course. In addition, task value was measured with six questions such as *It is important for me to learn the course material* and *I think the course material in this class is useful for me to learn*. (Cronbach’s alpha = 0.922) [39]. These items together created a scale of good fit (Chi-sq/df = 949.427, *p*< .001; RMSEA = 0.157; SRMR = 0.055; CFI = 0.995) [40]. Similar to perceptions of learning (Table 3 panel A), panel B indicates that students in active learning classrooms were 0.16 SD units less likely to agree that the course material is useful or important to their learning than students in lecture-based classrooms (*p* < 0.05). These estimates show the association between active learning on outcomes accounting for all possible mediational pathways (i.e., total effect). As previously discussed, the learning experience can be vastly different for students from minoritized populations. We were curious as to whether the above findings—that both perceptions of learning and task value were more negative for students in active learning courses—were more or less pronounced for racially minoritized students. Our analyses highlighted that minoritized students were just as likely to report decreased perceptions of learning and task value as represented students. These results can be found in S1 Table.

**Table 3:** Total, indirect (mediation), and direct effect estimates.

	Estimate
***Panel A*. *Feelings of Learning ***	
Indirect Effect (Mediation)	0.021***
Average Direct Effect	-0.116***
Total Effect	-0.095***
***Panel B*. *Task Value***	
Indirect Effect (Mediation)	0.059***
Average Direct Effect	-0.217**
Total Effect	-0.158*
***Panel C*. *Mediator ***	
Active Learning Instruction	0.15*

All of the regression estimates presented in this table include covariates to account for baseline differences among students in high group activity classroom versus low group activity classroom. High group activity classroom is identified using COPUS observation data.

*** *p* < 0.001

** *p* < 0.01

* *p* < 0.05.

To assess whether these negative relationships are driven by lower grades earned in active learning classrooms, we compare grade outcomes of students in active learning versus those in lecture-based instruction. Raw differences suggest that students in active-learning courses perform higher than lecture-based courses (3.15 in lecture-based versus 3.31 in active-learning), but this difference is not statistically significant with the inclusion of covariates. Thus, we conclude that students received similar grades in the class irrespective of whether the course was lecture-based or active learning, confirming previous findings that students taught with active learning approaches tend to be inaccurate in their perceptions of learning relative to students taught with the more traditional lecture approach (*B* = 0.006; *p* = 0.839) (S2 Table). Moreover, the relationship between students’ perceptions and active learning were independent of grades earned in the course (S3 Table).

### Students’ perceptions of effective instructor facilitation

Next, we examine the extent to which students’ perceptions of instructor effectiveness in facilitating group activities explain the negative relationship between active learning instruction and perceptions of learning and task value. Our mediating variable is a measure of students’ perceptions of instructor effectiveness in facilitating group activities and was measured with five questions such as: *the instructor clearly explained the purpose of the activity* and *encouraged students to engage with the activity through their demeanor*. These items were drawn from the student buy-in survey on active learning strategies (Cronbach’s alpha = 0.879) [41], and together created a scale of good fit (Chi-Sq(2) = 3.767, p = 0.152; SRMR = 0.006; RMSEA = 0.014; CFI = 1.00) [40]. Students in active learning classrooms were 0.15 SD units more likely to perceive that the instructor was effective at facilitating group activities than students in lecture-based classrooms (Table 3 panel C). Controlling for students’ perceptions of instructor effectiveness in facilitating group activities, the magnitude of the negative relationship on perceptions of learning increases from 9.5 to 12 percentage points. We interpret this estimate as the component of the total effect that does not occur through the perceptions of instructor effectiveness in facilitating group activities (i.e., holding constant students’ perceptions of effective instructor facilitation). Similarly, the magnitude of the negative relationship between instruction type and task value increases from -0.16 SD to -0.22 SD. The indirect effects shown under Table 3 panels A and B are all positive, indicating that students’ positive perceptions of instructors’ facilitation associated with active learning suppressed the overall negative effect. For instance, the point estimate of 0.02 in panel B suggests that students’ perceptions of learning in the course would have been about 2 percentage points more negative had students perceived instructor facilitation unfavorably in classrooms with high group activity (*p* < 0.001). Similarly, students’ reported task value would have been more negative by 0.06 SD units had it not been for their positive perceptions of instructor facilitation in active learning classes (*p* < 0.001).

### Moderated mediation analysis

We next examined whether the mediating influence differed depending on students’ racial identification (Table 4). We find that the interaction between the mediator and the racially minoritized identification are statistically indistinguishable from zero. Column 1 indicates that racially minoritized students—in active learning and lecture-based courses—did not perceive instructor facilitation of group activities any differently from represented students. Furthermore, the non-significant interaction effects between active learning and racially minoritized students, shown in Column 2 panels A and B, suggest that the relationship between active learning and perceptions of learning or task value do not vary by students’ racial identification. These findings indicate that the current mediational pathway is just as consequential for represented students as for racially minoritized students.

**Table 4:** Moderated mediation results.

	On Mediator	Total Effect	Direct Effect
***Panel A*. *Perceptions of Learning***			
Active Learning	0.161*	-0.090***	-0.117***
	(0.059)	(0.017)	(0.018)
Racially Minoritized (RM)	0.031	0.036*	0.136
	(0.052)	(0.015)	(0.067)
Active Learning x RM	-0.021	-0.008	0.001
	(0.062)	(0.028)	(0.029)
Perceptions of Instructor Facilitation			0.151***
			(0.026)
Perceptions of Instructor Facilitation x RM			-0.027
			(0.016)
R^2^	0.108	0.064	0.150
N	4257	4257	4257
***Panel B*. *Task Value***			
Active Learning		-0.101	-0.164*
		(0.060)	(0.062)
RM		0.064	0.266*
		(0.055)	(0.128)
Active Learning x RM		-0.070	-0.049
		(0.069)	(0.061)
Perceptions of Instructor Facilitation			0.359***
			(0.055)
Perceptions of Instructor Facilitation x RM			-0.054
			(0.030)
R^2^		0.143	0.270
N		4257	4257

Each panel in columns 2 and 3 represent different regression results. Column 1 estimates the differential effect on the mediator by race. Column 2 estimates moderation of the overall treatment effect. Column 3 estimates the moderation of the treatment effect by race accounting for differential effect on the mediator.

*** *p* < 0.001

** *p* < 0.01

* *p* < 0.05.

### Triangulating students’ perceptions of effective instructor facilitation

Having observed the positive mediating effect of students’ perceptions of instructor effectiveness in facilitating group activities, we triangulated whether students’ perceptions of instructors’ facilitation of group activities match that of the instructors’. If students and instructors are misaligned in what is occurring in the classroom, students may report decreased utility for in-class activities and perceive that they are learning less. Previous literature documented a misalignment between what the instructor believed had occurred in the classroom versus what the students believed happened [41, 42]. Despite instructors’ best intentions, students may express decreased perceptions of learning and task value when there exists a misalignment between what the instructor believed had occurred in the classroom versus the students.

**Fig 2:**
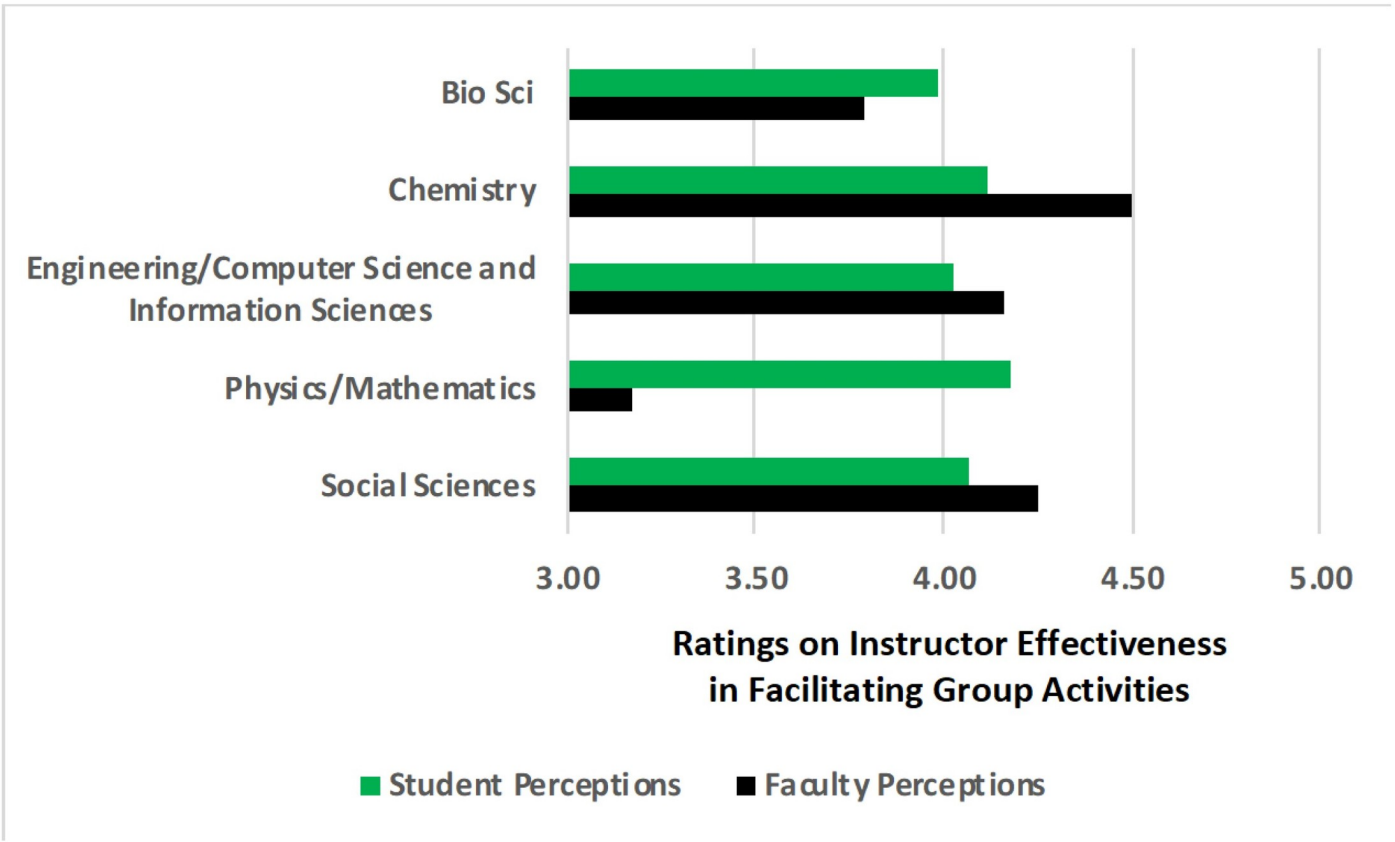
Alignment of faculty and student perceptions across disciplines. Social Science includes public policy/social ecology/psychology.

We assessed the level of alignment between instructors and students in their responses to identical survey questions related to effectiveness of facilitating group activities. We first conduct a simple correlation between instructor and students’ responses to perceptions of instructor facilitation and find essentially no correlation (S1 Fig). In addition, this misalignment in perception of instructor effectiveness varied widely across disciplines (Fig 2). Whereas students were more positive about instructor facilitation in Biological Sciences and Mathematics/Physics than instructors themselves, instructors were more positive about their facilitation than students in Chemistry, Engineering/Computer Science, Public Policy, Psychology, and Social Ecology. These results indicate that there exists a misalignment in perceptions of effective instructor facilitation between instructors and students, and that the directionality of these misalignments differ depending on the discipline. These findings suggest that instructors may generally be unaware of when students perceive that their facilitation was effective or ineffective.

We also verify that students’ survey responses regarding the occurrence of classroom activities align with independent observations of classroom practices (COPUS). We corroborate students’ responses to questions regarding *frequency of lecturing* and *frequency of group activities* to our COPUS results and found that students and independent observers were closely aligned with one another (S4 Table). So while there is clear alignment between students’ perceptions of classroom activities and the actual activities themselves (as measured by independent classroom observers), students’ perceptions of instructor effectiveness do not align with faculty’s self-rating of how effective they were in facilitating these same group activities.

## Discussion and conclusions

Over the past few decades, there has been heightened interest in increasing the use of active learning pedagogies in higher education settings [4, 7, 10, 43], yet wide-scale implementation has yet to occur [12]. This study found that students of all races in active learning classrooms perceived they learned less and rated the utility of the course activities lower than their peers in more lecture-based courses, despite earning similar grades. Our mediation analysis suggests that the relationship between active learning and perceptions of learning and task value would have been even more negative had it not been for students’ positive perceptions of instructor facilitation in active learning classrooms. In subsequent analyses, we investigated whether the instructors and students were aligned in their assessment of effective instructor facilitation and found little alignment.

Our results suggest that effective instructor facilitation not only influences students’ learning in the course [26] but also students’ self-assessment of learning and perceived utility of the learning activities. As such, instructors should be systematic and intentional in facilitating group activities, particularly as there can exist a disconnect between what students and faculty perceive is happening in the classroom [42]. Potential means through which instructors can accomplish improved facilitation fall under the umbrella of pedagogical competence, which includes giving clear and relevant group assignments, giving direct feedback to students, and facilitating group discussions [44]. For example, instructors may want to discuss the broader purpose and expectations before every in-class group activity, walk around the classroom during the activities, and provide feedback and answer questions, as recommended by previous research on effective teaching and pedagogical competence [44].

While the mediator was just as consequential for racially-minoritized students as for represented students, faculty should continue to interrogate existing practices and be mindful of different racial group dynamics when facilitating group activities, given prior literature that racially minoritized students may experience group activities differently compared to represented students [19, 20]. Indeed, previous studies examining the ways in which students’ identities intersect with active learning instructional approaches showed that minoritized students tend to feel less comfortable in whole-group discussions relative to small group discussions [19] and that minoritized students tend to prefer conducting group work with those who share their identities [20]. Given this research, faculty should be mindful of how they group students (i.e., ensuring that racially minoritized students are in a group with at least one other racially minoritized student). In fact, the nuances of active learning approaches may explain why the correlation between instructor facilitation and student perceptions of learning did not differ among racially minoritized students. Future research that examines the intersection between racial/ethnic identities and various active learning approaches is needed given that active learning environments provide an opportunity to elevate sense of belonging among minoritized students [20].

Without training on effective group facilitation, faculty may operate with the incorrect assumption that their in-class group facilitation is effective. Accordingly, it is important that faculty remain transparent in their pedagogical decisions and that students feel personally invested in the activities. To address this misalignment, institutions should consider offering faculty the opportunity to learn about these evidence-based instructional practices through active learning training [45, 46]. The burden of clearly explaining the activities’ purpose may decrease in an institutional environment where active learning becomes the norm. As more faculty are provided with institutionalized support to implement active learning, students may also shift their perceptions on active learning and gradually buy-in to these course activities.

A limitation of this study is that we are comparing a variety of STEM courses across the 25 courses included in the study sample and thus are not able to make direct comparisons of similar courses taught in active learning versus lecture formats. This means that course assessments, grading practices, and instructional practices vary between and within courses. For instance, specific in-class group activities are likely different depending on the scope of the course and/or the instructor. Furthermore, to better understand student perceptions of instructor effectiveness, it will be important to distinguish the specific types of group activities that instructors have facilitated across the 25 STEM classrooms and whether certain activities are more positively perceived than others by students. In some instances, group problem solving activities may be a significant fraction of a lecture period whereas other cases may leverage clicker questions as a brief means to summarize a lecture. Identifying student perceptions of their instructor’s effectiveness at facilitating different activities may help us understand which contribute disproportionately to student feelings of learning or which should be emphasized during professional development programs. A related limitation to the work is that the COPUS protocol illustrates what the instructor and students are physically doing within a course period, but does not characterize the content of these classroom interactions. Follow up work may leverage the Classroom Discourse Observation Protocol (CDOP) to identify whether particular types of instructor discourse correlate with negative or positive student perceptions of instructor effectiveness [47].

It is important to note that instruction that incorporates high levels of group activities may be seen by students as more active, engaging, and *difficult*. Many college students erroneously associate easy and enjoyable tasks to mean that they are learning the material while associating effortful and difficult tasks as the lack thereof [48, 49]. Yet, as students engage with a challenging learning process, they retain the material longer and understand the concept more deeply [48]. Given that students, on average, tend to feel like they learned less in active learning classrooms, faculty should, as part of their role as facilitators, clarify to students throughout the course that effortful learning leads to greater mastery in the long-run and immediate fluency does not necessarily equate to mastery. And that when students learn to embrace a more active and engaging learning environment, they may come to realize that they have learned less superficially and have gained skills and social networks that contribute to thriving academically.

## Supporting information

S1 Fig Scatterplot between faculty and student perceptions of instructor facilitation of group activities. (PDF)

S1 Table Interaction effects by racially minoritized students and represented Students. (PDF)

S2 Table Relationship between active learning and course grades. (PDF)

S3 Table Relationship between active learning and measures by levels of course grades. (PDF)

S4 Table Alignment between COPUS results and student survey response. (PDF)

S5 Table Total, indirect, and direct effect estimates accounting for type I error rate. (PDF)

S1 Appendix List the survey questions fielded to students. (DOCX)
