# Supplementary material for: Instructor facilitation mediates students’ negative perceptions of active learning instruction
Source: PLoS One. 2021 Dec 23;16(12):e0261706. doi: 10.1371/journal.pone.0261706 (PMC8699631; doi:10.1371/journal.pone.0261706)

**Fig S1. Scatterplot between faculty and student perceptions of instructor facilitation of group activities.**

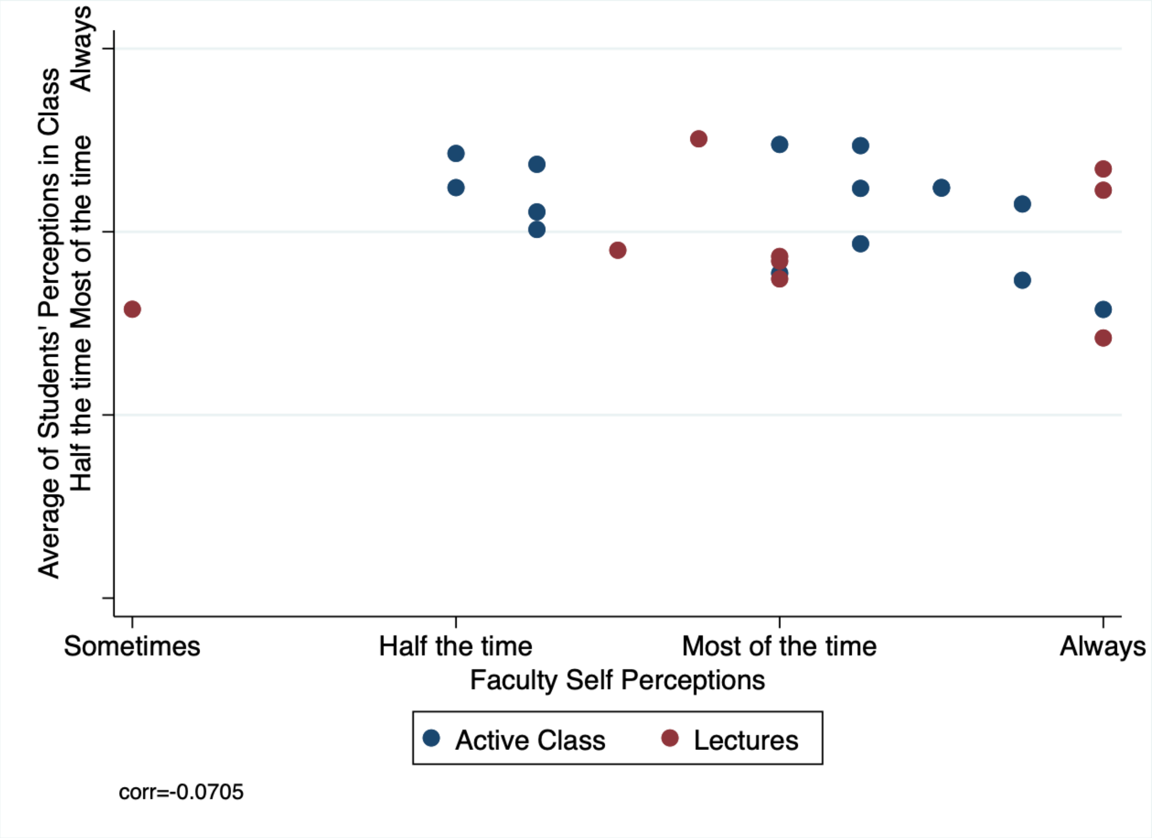

Supplement: S1 Fig — (PDF) [file pone.0261706.s001.pdf]
