# Supplementary material for: Instructor facilitation mediates students’ negative perceptions of active learning instruction
Source: PLoS One. 2021 Dec 23;16(12):e0261706. doi: 10.1371/journal.pone.0261706 (PMC8699631; doi:10.1371/journal.pone.0261706)
Supplement: S1 Table — (PDF) [file pone.0261706.s002.pdf]

Supplementary Information

Instructor facilitation mediates students' negative perceptions of active learning instruction

Table S1. Interaction effects by racially minoritized students and represented Students. In this analysis, we include student-level covariates, classroom-level covariates, instructor characteristics, entry term fixed effects, time trend, and department fixed effects. Standard errors are in parentheses.

	Perceptions of Learning	Task Value
Active Learning	-0.112***	-0.182*
	(0.018)	(0.074)
Racially Minoritized	0.032+	0.064
	(0.017)	(0.056)
Active Learning x Racially Minoritized	-0.005	-0.075
	(0.029)	(0.071)
Perception of Instructor Effectiveness in Facilitating Group Activities	0.136***	0.389***
	(0.025)	(0.064)
R-sq	0.150	0.269
N	4257	4257

+ $p < 0.10$, * $p < 0.05$, ** $p < 0.01$, *** $p < 0.001$
