# Supplementary material for: Instructor facilitation mediates students’ negative perceptions of active learning instruction
Source: PLoS One. 2021 Dec 23;16(12):e0261706. doi: 10.1371/journal.pone.0261706 (PMC8699631; doi:10.1371/journal.pone.0261706)
Supplement: S2 Table — (PDF) [file pone.0261706.s003.pdf]

Table S2. Relationship between active learning and course grades. In this analysis, we include student-level covariates, classroom-level covariates, instructor characteristics, entry term fixed effects, time trend, and department fixed effects. Standard errors are in parentheses.

	Grades
Active Learning vs. Lecture-Based	0.006
	(0.028)
Racially Minoritized	-0.053*
	(0.026)
Perception of Instructor Facilitation of Group Activities	0.023
	(0.022)
R-sq	0.484
N	4257

+ p < 0.10, * p < 0.05, ** p < 0.01, *** p < 0.001
