# Supplementary material for: Instructor facilitation mediates students’ negative perceptions of active learning instruction
Source: PLoS One. 2021 Dec 23;16(12):e0261706. doi: 10.1371/journal.pone.0261706 (PMC8699631; doi:10.1371/journal.pone.0261706)
Supplement: S3 Table — (PDF) [file pone.0261706.s004.pdf]

Table S3. Relationship between active learning and measures by levels of course grades. In all four models, we include student-level covariates, classroom-level covariates, instructor characteristics, entry term fixed effects, time trend, and department fixed effects. Standard errors are in parentheses.

	(1)<br>Perceptions of Learning	(2)<br>Perceptions of Learning x Grades	(3)<br>Task Value	(4)<br>Task Value x Grades
Active Learning	-0.115***	-0.045	-0.221**	-0.023
	(0.016)	(0.074)	(0.065)	(0.195)
Perception of Instructor Facilitation of Group Activities	0.135***	0.135***	0.385***	0.386***
	(0.024)	(0.024)	(0.060)	(0.061)
Course Grades	0.065***	0.074***	0.209***	0.236***
	(0.010)	(0.012)	(0.030)	(0.042)
Perception of Instructor Facilitation of Group Activities x Grades		-0.021		-0.060
		(0.022)		(0.059)
R-sq	0.160	0.160	0.288	0.289
N	4257	4257	4257	4257

+ $p < 0.10$, * $p < 0.05$, ** $p < 0.01$, *** $p < 0.001$
