# Supplementary material for: Instructor facilitation mediates students’ negative perceptions of active learning instruction
Source: PLoS One. 2021 Dec 23;16(12):e0261706. doi: 10.1371/journal.pone.0261706 (PMC8699631; doi:10.1371/journal.pone.0261706)
Supplement: S4 Table — (PDF) [file pone.0261706.s005.pdf]

Table S4. Alignment between COPUS results and student survey response

Student Survey Item	Lecture-Based	Active Learning
Spent most of time lecturing		
Never	1.1	9.9
Sometimes	2.7	16.2
About half the time	7.7	13.9
Most of the time	35.3	30
Always	53.2	30.1
Total	1902	2355
Pearson chi2(4) = 507.9807, $p < 0.001$		
Discuss with 2 or more an activity		
Never	36.1	16.6
Sometimes	23.3	15.2
About half the time	12.6	14.3
Most of the time	11	23.9
Always	17	30.1
Total	1902	2355
Pearson chi2(4) = 369.1614, $p < 0.001$		
