# Supplementary material for: Instructor facilitation mediates students’ negative perceptions of active learning instruction
Source: PLoS One. 2021 Dec 23;16(12):e0261706. doi: 10.1371/journal.pone.0261706 (PMC8699631; doi:10.1371/journal.pone.0261706)
Supplement: S5 Table — (PDF) [file pone.0261706.s006.pdf]

Table S5. Total, indirect, and direct effect estimates accounting for Type I error rate. All the regression estimates presented in this table include covariates to account for baseline differences among students in high group activity classroom versus low group activity classroom. High group activity classroom is identified using COPUS observation data. The Sharpened q-values tests for the extent to which the statistical significance may be a false rejection (Type I error rate).

	Estimate	p-value	Sharpened q-values
Panel A. Feelings of Learning			
Indirect Effect (Mediation)	0.021	0.0001	0.0007
Average Direct Effect	-0.116	0.0001	0.0007
Total Effect	-0.095	0.0001	0.0007
Panel B. Task Value			
Indirect Effect (Mediation)	0.059	0.0001	0.0007
Average Direct Effect	-0.217	0.002	0.006
Total Effect	-0.158	0.024	0.03
Panel C. Mediator			
Active Learning Instruction	0.15	0.014	0.03
