# Supplementary material for: Instructor facilitation mediates students’ negative perceptions of active learning instruction
Source: PLoS One. 2021 Dec 23;16(12):e0261706. doi: 10.1371/journal.pone.0261706 (PMC8699631; doi:10.1371/journal.pone.0261706)
Supplement: S1 Appendix — (DOCX) [file pone.0261706.s007.docx]

**S1 Appendix. List the survey questions fielded to students**

| *Perceptions of instructor effectiveness in facilitating group work* | | |  |  |  |
| --- | --- | --- | --- | --- | --- |
| Thinking about the group activities you have done in this class, please indicate how often your instructor did the following: | | | | | |
|  | Never (1) | Sometimes (2) | About half the time (3) | Most of the time (4) | Always (5) |
| Clearly explained the purpose of activities we did in class | o | o | o | o | o |
| Discussed how activities related to my learning | o | o | o | o | o |
| Clearly explained what I was expected to do for activities in class | o | o | o | o | o |
| Encouraged us to engage with activities through his/her demeanor | o | o | o | o | o |

*Perceptions of learning*

| Based on your experiences in this class, please indicate how much you agree or disagree with the following statements. | | | | | |
| --- | --- | --- | --- | --- | --- |
|  | Strongly disagree (1) |  |  |  | Strongly agree (5) |
| I feel like I learned a great deal from this course. | o | o | o | o | o |

*Task Value*

| Please indicate to what degree you feel the following statements are true: | Definitely false (1) | Probably false (2) | Neither true nor false (3) | Probably true (4) | Definitely true (5) |
| --- | --- | --- | --- | --- | --- |
| I will be able to use what I learn in this course in other courses. | o | o | o | o | o |
| It is important for me to learn the course material in this class. | o | o | o | o | o |
| I am very interested in the content area of this course. | o | o | o | o | o |
| I think the course material in this class is useful for me to learn. | o | o | o | o | o |
| I like the subject matter of this course. | o | o | o | o | o |
| Understanding the subject matter of this course is very important to me. | o | o | o | o | o |
|  |  |  |  |  |  |

**List the survey questions fielded to faculty**

| Thinking about the group activities you have facilitated in this class, please indicate how often you did the following:   \|  \| Never (1) \| Sometimes (2) \| About half the time (3) \| Most of the time (4) \| Always (5) \| \| --- \| --- \| --- \| --- \| --- \| --- \| \| Clearly explained the purpose of activities we did in class \| o \| o \| o \| o \| o \| \| Discussed how this activity related to student learning \| o \| o \| o \| o \| o \| \| Clearly explained what students were expected to do for the activity \| o \| o \| o \| o \| o \| \| Encouraged students to engage with the activity \| o \| o \| o \| o \| o \| |
| --- | --- | --- | --- | --- | --- | --- | --- | --- | --- | --- | --- | --- | --- | --- | --- | --- | --- | --- | --- | --- | --- | --- | --- | --- | --- | --- | --- | --- | --- | --- |
